# CD19^+^CD24^hi^CD38^hi^Bregs involved in downregulate helper T cells and upregulate regulatory T cells in gastric cancer

**DOI:** 10.18632/oncotarget.5588

**Published:** 2015-09-10

**Authors:** Weiwei Wang, Xiangliang Yuan, Hui Chen, Guohua Xie, Yanhui Ma, Yingxia Zheng, Yunlan Zhou, Lisong Shen

**Affiliations:** ^1^ Department of Clinical Laboratory, Xinhua Hospital, affiliated to Shanghai Jiao Tong University School of Medicine, Shanghai, China

**Keywords:** gastric cancer, regulatory B cells, IL-10, TGF-β, regulatory T cells, immune escape

## Abstract

Regulatory B cells (Bregs) play a critical role in inflammation and autoimmune disease. We characterized the role of Bregs in the progression of gastric cancer. We detected an increase in Bregs producing IL-10 both in peripheral blood mononuclear cells (PBMCs) and in gastric tumors. Multicolor flow cytometry analysis revealed that a subset of CD19^+^CD24^hi^CD38^hi^ B cells produces IL-10. Functional studies indicated that increased Bregs do not inhibit the proliferation of CD3^+^T cells or CD4^+^ helper T cells (Th cells). However, Bregs do suppress the secretion of IFN-γ and TNF-α by CD4^+^Th cells. CD19^+^CD24^hi^CD38^hi^Bregs were also found to correlate positively with CD4^+^FoxP3^+^ regulatory T cells (Tregs). Neutralization experiments showed that Bregs convert CD4^+^CD25^−^ effector T cells to CD4^+^FoxP3^+^Tregs via TGF-β1. Collectively, these findings demonstrate that increased Bregs play a immunosuppressive role in gastric cancer by inhibiting T cells cytokines as well as conversion to Tregs. These results may provide new clues about the underlying mechanisms of immune escape in gastric cancer.

## INTRODUCTION

Gastric cancer is among the most common malignant tumors of the digestive tract [1]. Although the rates of gastric cancer have decreased substantially in most parts of the world, mortality is still high globally, and gastric cancer is ranked fourth in incidence among cancers [2, 3], particularly in less-developed countries [4]. Eastern Asia has the highest incidence of gastric cancer occurs [2]. In addition to *H. pylori* infection and poor dietary habits, immune regulation also plays an important role in gastric cancer development, progression, metastasis, and resistance to treatment. Our previous studies found that immunosuppressive cells, especially immunosuppressive regulatory T cells (Tregs), play important roles in tumor escape in gastric cancer [5-7].

In addition to Tregs, there is also a discrete subset of B cells, described and confirmed as regulatory B cells (Bregs) [8-10]. However, there are no specific markers for Bregs [11, 12]. Studies in mouse models have reported regulatory functions for different B cell subsets, such as CD19^+^IL-10^+^ [13], CD19^+^CD5^+^CD1d^hi^ [14], CD5^+^CD19^+^B220^low^ [15] and CD19^+^CD25^+^CD1d^hi^ IgM^hi^CD5^−^CD23^−^Tim-1^−^ [16]. Other B cell subsets, such as CD19^+^FSC^high^ [17], CD19^+^CD5^+^IL-10^+^ [18], CD19^+^CD5^+^Foxp3^+^ [19], CD19^+^CD1d^hi^CD5^+^ [20], CD19^+^CD24^hi^CD38^hi^ [21-23], CD19^+^CD24^hi^CD27^+^ [24, 25] and granzyme B^+^ cells [26], play regulatory roles in human diseases. As there is no agreed consensus regarding the combination of Breg cell-linked markers, various research teams have been identifying Breg cells using a diverse array of markers. As Breg cell function and cell sorting depend on the type and number of markers used, the most appropriate markers for Breg cells in human gastric cancer need confirmation.

Emerging evidence suggests that Bregs play essential roles in inflammation and autoimmune diseases, such as experimental autoimmune encephalomyelitis (EAE) [27], systemic lupus erythematosus (SLE) [21], rheumatoid arthritis (RA) [22], multiple sclerosis (MS) [28], inflammatory bowel disease (IBD) [16, 29], hematological diseases [23, 30], parasitic infections [31, 32], tuberculosis [20, 33] and graft versus host disease [18, 34]. Although Bregs have been extensively studied in these diseases, there is little knowledge on the role of Bregs in human cancer. It is reported that GrB-expressing B cells (granzyme B^+^ Bregs) reside within the microenvironment of different tumor types [35]. In mice, tumor cells can induce B cells to produce IL-10, which inhibits CD8^+^T cells activity and reduces IFN-γ production by CD8^+^T and NK cells. IL-10^+^ Breg deficiency can enhance anti-tumor action [36], while Bregs evoked by tumor cells (tBregs) inhibit anti-tumor responses and upregulate Tregs, thus facilitating breast cancer metastasis [37]. Tumor metastasis can also be abrogated by the inactivation of tBregs in mice [38]. While experimental models have yielded important insights into the mechanisms by which B cells affect tumor immunity, the role of Bregs in human gastric cancer has not been previously described.

In this study, we quantified CD19^+^B cell numbers in peripheral blood mononuclear cells (PBMCs), peritumoral tissues, and tumor tissues, and detected the frequency of CD19^+^CD24^hi^CD38^hi^Bregs in gastric cancer. We found that CD24^hi^CD38^hi^Bregs inhibited the expression of inflammatory cytokines produced by CD4^+^T cells. In addition, using an *in vitro* co-culture system, we found that CD19^+^CD24^hi^CD38^hi^ Bregs induced the conversion of CD4^+^CD25^−^ effector T cells to CD4^+^FoxP3^+^Tregs. This conversion depended upon TGF-β1 but not IL-10. Our results suggest that CD19^+^CD24^hi^CD38^hi^ Bregs are involved in immunosuppression in gastric cancer via inhibition of anti-tumor helper T cells (Th1 cells) and promotion of pro-tumor Treg cells. To our knowledge, this study is the first to define the role and mechanism of action of Bregs in human gastric cancer.

## RESULTS

### Increased IL-10-producing Breg cells in gastric cancer

**Figure 1:**
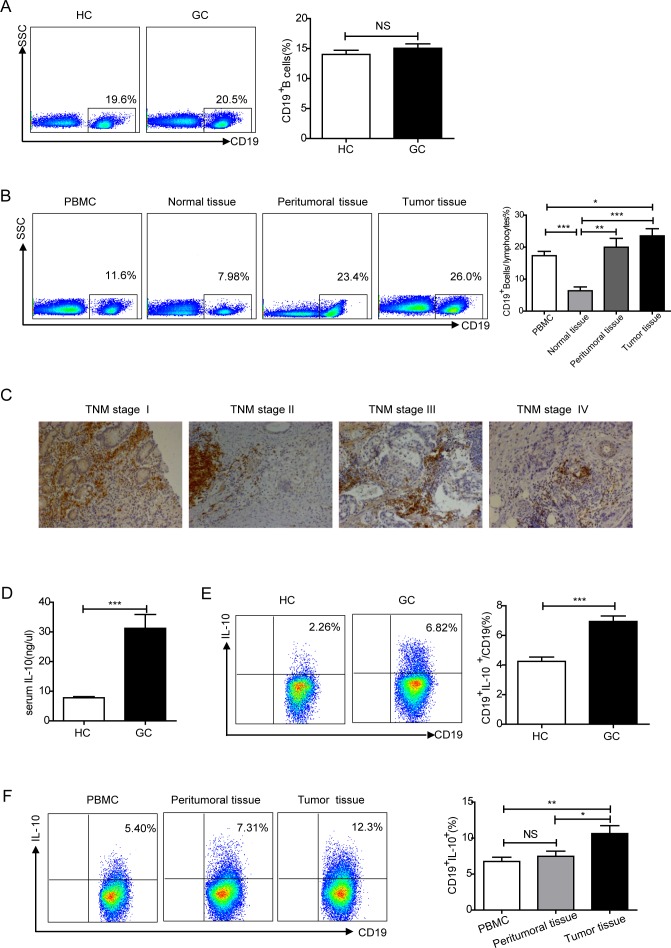
Analysis of the percentage of B cells and IL-10-producing Breg cells in gastric cancer **A.** Representative flow cytometry plot and graph showing the percentage of CD19^+^ B cells among CD45^+^ lymphocytes from healthy controls (HC, *N* = 40) and gastric cancer patients (GC, *N* = 107) (NS, no statistical differences). **B.** Flow cytometry analysis of the percentage of CD19^+^ B cells among lymphocytes in PBMCs from GCs and single cell suspensions of normal tissues, peritumoral tissues and tumor tissues (*N* = 45). There is an increased percentage of CD19^+^B cells in tumor tissues (*P < 0.05, **P < 0.01, ***P < 0.001). **C.** Representative immunohistochemical photos showing the distribution of CD19^+^B cells in paraffin-embedded tumor tissues of different TNM stages. **D.** ELISA detecting IL-10 from HC serum (*N* = 10) and GC serum (*N* = 40) (***P < 0.001). E. Percentage of CD19^+^IL-10^+^cells among CD19^+^B cells in HC (*N* = 40) and GC (*N* = 45) samples (***P < 0.001). F. Representative flow cytometry plot depicting the percentage of CD19^+^IL-10^+^cells among CD19^+^B cells in PBMCs of GCs (*N* = 10) and single cell suspensions of peritumoral and tumor tissues (*N* = 10). The graph shows the increased percentage of CD19^+^IL-10^+^ cells in the tumor tissues of GCs (NS, no statistical differences, **P < 0.01).

As B lymphocyte cells correlate with many significant functions in immune homeostasis [39, 40], we measured the percentage of CD19^+^B cells among CD45^+^ lymphocytes in peripheral blood from healthy controls (HCs) and gastric cancer patients (GCs) via flow cytometry. There was no statistical difference between HCs and GCs (*P* > 0.05, Figure 1A). Lymphocyte infiltration into solid tumors is an important factor in prognosis [40]. Thus, to explore the characteristics of B cells in patients with gastric cancer, the percentage of CD19^+^B cells was analyzed in PBMCs, normal tissues, peritumoral tissues and tumor tissues using flow cytometry. When compared with normal tissues or PBMCs, the percentage of CD19^+^ B cells was higher in peritumoral and tumor tissues (*P* < 0.001 or *P* < 0.05, Figure 1B). Immunohistochemical analyses of CD19^+^ B cells revealed a large number of B cells in tumor tissues of different TNM stages, suggesting the infiltration of B cells (Figure 1C, Figure S1A).

Emerging evidence has shown an increased percentage of IL-10^+^Bregs in numerous diseases [9, 41]. IL-10 levels were measured in serum from both HCs and GCs. GCs had increased serum IL-10 as compared to HCs (*P* < 0.001, Figure 1D). IL-10-producing B cells were also analyzed in PBMCs from HCs and GCs, the percentage of CD19^+^IL-10^+^ cells of CD19^+^B cells was increased in GCs compared to HCs (*P* < 0.001, Figure 1E). To further confirm this characteristic in tumor tissues, we performed fluorescence labeling and confocal microscopy analyses of CD19^+^IL-10^+^B cells were performed in tumors (Figure S1B). IL-10^+^ B cells were also analyzed in PBMCs, peritumoral tissues, and tumor tissues of gastric cancer patients using flow cytometry. Compared with PBMCs and peritumoral tissues, there was an increase in IL-10-producing Bregs in tumor tissues (*P* < 0.01 or *P* < 0.05, Figure 1F).

### Characterization of IL-10-producing Breg cells in gastric cancer

**Figure 2:**
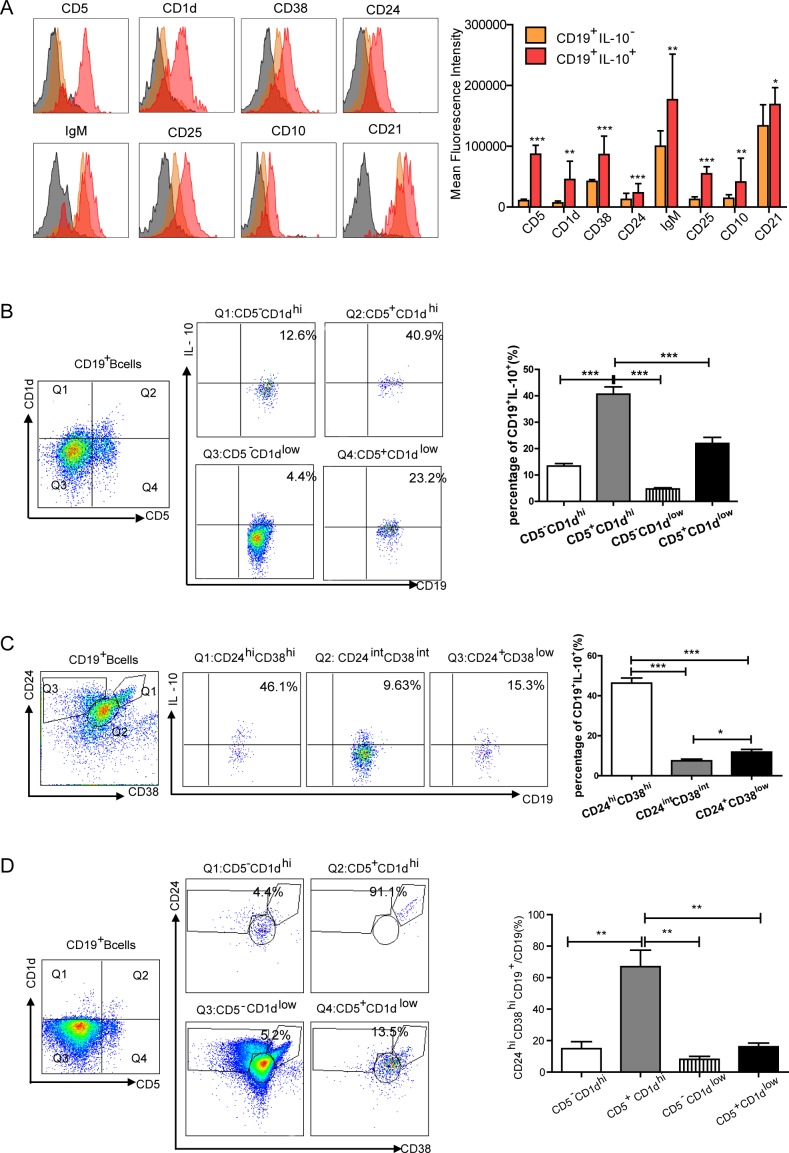
Multicolor flow cytometry analysis of the characterization of IL-10-producing Bregs in gastric cancer **A.** The phenotype of IL-10-producing Bregs was analyzed using flow cytometry (gray area is isotype). The histogram depicts mean fluorescence intensity (MFI) of different cell surface makers expressed in IL-10^+^ (red area) and IL-10^−^ (orange area) CD19^+^ B cells. Increased MFI was detected on IL-10^+^ B cells compared to IL-10^−^ B cells (*P < 0.05, **P < 0.01, ***P < 0.001). **B.** Representative flow cytometry plot showing the gating strategy for CD19^+^ B cells stained with CD5 and CD1d; four quadrants of CD5 and CD1d on IL-10-producing cells were analyzed. The graph shows that CD5^+^CD1d^hi^ was the main source of IL-10 (***P < 0.001). **C.** Flow cytometry analysis of IL-10-producing B cells stained with CD24 and CD38. The graph shows that CD24^hi^CD38^hi^ was the primary source of IL-10 (*P < 0.05, ***P < 0.001). **D.** Plot showing the correspondence between CD5^+^CD1d^hi^ and CD24^hi^CD38^hi^ cells. CD24^hi^CD38^hi^ contains the majority of CD5^+^CD1d^hi^ cells (**P < 0.01).

We next conducted a detailed surface marker analysis of IL-10^+^Breg cells. PBMCs from gastric cancer patients were stained with various combinations of monoclonal antibodies to proteins which have been suggested for use in identifying Bregs [12], including: CD5, CD1d, CD38, CD24, IgM, CD25, CD10, and CD21. Compared with IL-10^−^ B cells, IL-10^+^ B cells expressed higher levels of CD5, CD38, CD24 and CD25 (*P* < 0.001, Figure 2A). The majority of IL-10^+^ B cells also expressed higher level of CD1d, IgM and CD10 (*P* < 0.01, Figure 2A) and CD21 (*P* < 0.05, Figure 2A) when compared to IL-10^−^ B cells. The differences between IL-10^−^ and IL-10^+^ were also compared both in healthy control PBMCs and gastric cancer lesions (Figure S2A-B). As CD5/CD1d are regulatory facrors in mouse models of inflammation [14, 41] and CD24/CD38 are involved in human autoimmune diseases [23, 37], we assessed the expression of these markers in CD19^+^ B cells via flow cytometry. CD5^+^CD1d^hi^ cells and CD24^hi^CD38^hi^ cells were the main source supplies of IL-10 (41.3±5.89% of IL-10 gated on CD19^+^CD5^+^CD1d^hi^, 46.3±7.46% of IL-10 gated on CD19^+^CD24^hi^CD38^hi^, *P* < 0.001, Figure 2B-2C). Interestingly, the majority of CD5^+^CD1d^hi^ cells (67.6±19.62%) also expressed CD24^hi^CD38^hi^ (*P* < 0.01, Figure 2D).

### Increased CD19^+^CD24^hi^CD38^hi^Bregs is positively correlated with Tregs in gastric cancer

**Figure 3:**
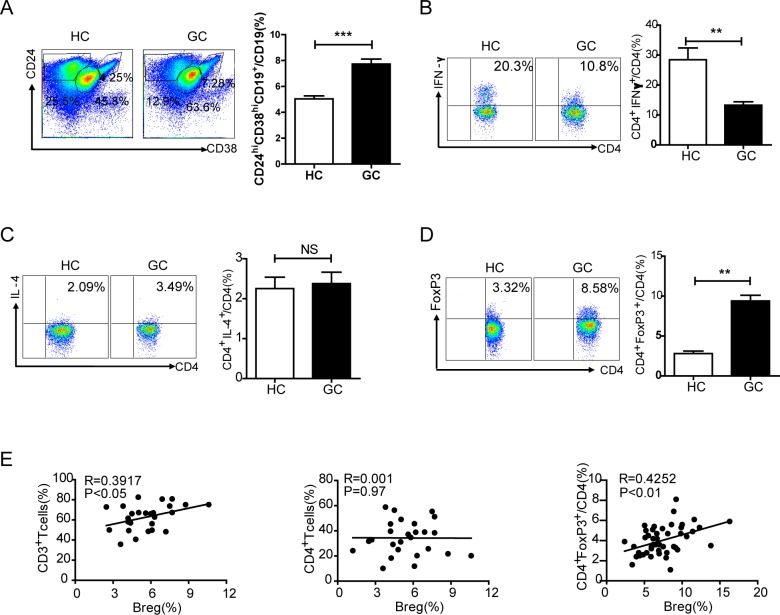
Correlations between CD24^hi^CD38^hi^Bregs and other T cell subsets in gastric cancer **A.** Representative flow cytometry plot depicting the gating strategy for CD19^+^CD24^hi^CD38^hi^Bregs, CD19^+^CD24^int^CD38^int^ and CD19^+^CD24^+^CD38^low/−^ B cells in PBMCs of healthy controls (HC, *N* = 40) and gastric cancer patients (GC, *N* = 45). The graph shows a significantly increased percentage of Bregs in GC (***P < 0.001). **B.** Flow cytometry analysis of the percentage of CD4^+^IFN-γ^+^ Th1 cells among CD4^+^T cells in PBMCs from HC and GC (20.3% *vs*. 10.8%) (**P < 0.01). **C.** Plot showing the percentage of CD4^+^IL-4^+^Th2 cells from HC and GC (2.09% *vs*. 3.49%) (NS, no statistical differences). **D.** The percentage of CD4^+^FoxP3^+^Treg cells from HCs and GCs is analyzed (3.32% *vs*. 8.58%) (**P < 0.01). **E.** Graphs showing the correlation between CD19^+^CD24^hi^CD38^hi^Bregs and other T cell subsets in gastric cancer, a positive correlation between Bregs and CD3^+^T cells (*R* = 0.3917, *P* < 0.05), no correlation between Bregs and CD4^+^Th cells (*R* = 0.001, *P* = 0.97), and a positive correlation between Bregs and CD4^+^FoxP3^+^Tregs(*R* = 0.4252, *P* < 0.01).

Next we used flow cytometry to detect the percentage of CD19^+^CD24^hi^CD38^hi^Bregs among CD19^+^B cells in PBMCs of HCs and GCs. The percentage of Bregs in GCs was higher than that of HCs (7.5% *vs*. 5.0%, *P* < 0.001, Figure 3A). However, the percentage of Bregs was not correlated with TNM stage (Figure S3). To further understand the relationship among Bregs and other T cell subsets in gastric cancer, the percentages of CD3^+^T cells, CD4^+^Th cells, CD4^+^IFN-γ^+^Th1 cells, CD4^+^IL-4^+^Th2 cells and CD4^+^FoxP3^+^Treg cells were also detected in PBMCs from GCs. CD3^+^T cells constituted 75% of the CD45^+^ lymphocytes in PBMCs of GCs, while CD4^+^ Th cells constituted an average of 39% of the PBMCs (Figure S4). CD4^+^IFN-γ^+^Th1 cells were also detected via flow cytometry. GCs showed a decreased percentage of CD4^+^IFN-γ^+^Th1 cells as compared to HCs (*P* < 0.01, Figure 3B). However, there was no noticeable difference in CD4^+^IL-4^+^Th2 cells between HCs and GCs (*P* > 0.05, Figure 3C). To evaluate Treg cells, CD4^+^FoxP3^+^Treg cells were also analyzed using flow cytometry. CD4^+^FoxP3^+^Tregs made up 8.58% of CD4^+^T cells in GCs compared to 3.32% in HCs (*P* < 0.01, Figure 3D). Linear regression revealed no correlation between Bregs and CD4^+^Th cells, whereas there were positive correlations between Bregs and CD3^+^T cells (*R* = 0.3917, *P* < 0.05) and Bregs and Tregs (*R* = 0.4252, *P* < 0.01, Figure 3E).

### CD19^+^CD24^hi^CD38^hi^ Bregs inhibit cytokine production in CD4^+^Th cells

**Figure 4:**
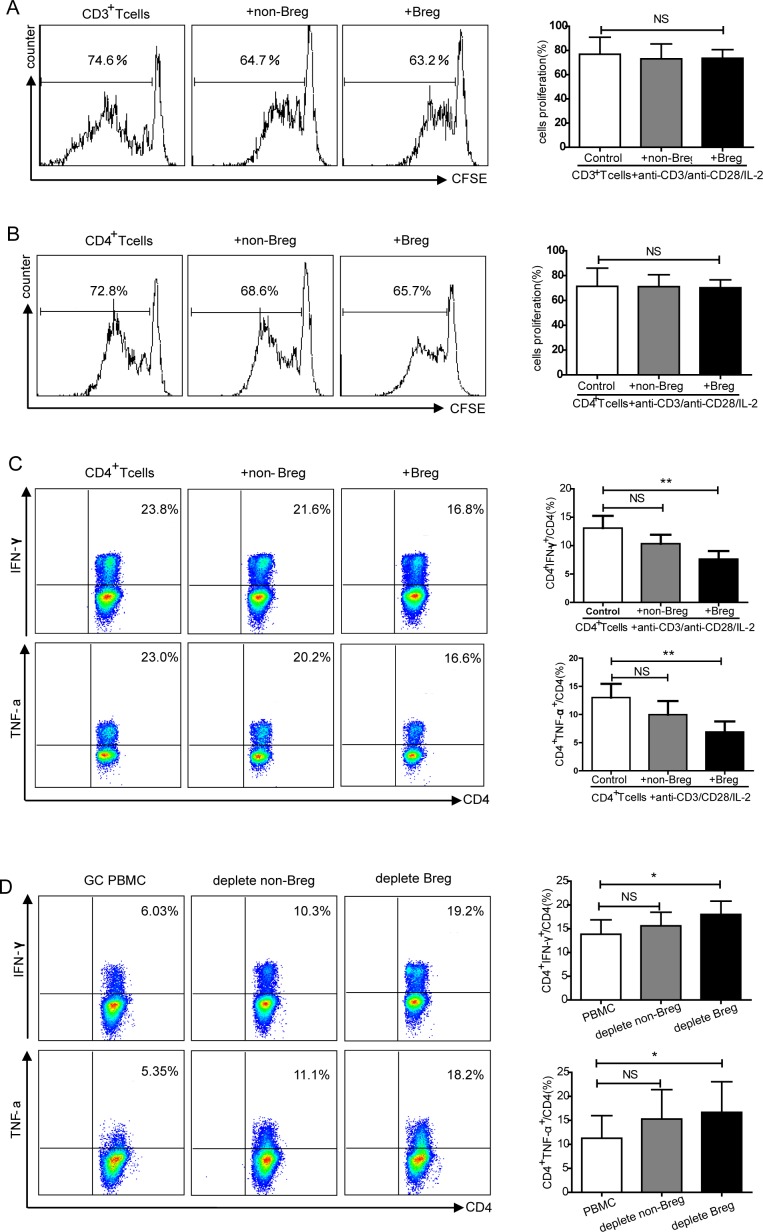
Functional analysis of CD19^+^CD24^hi^CD38^hi^Breg regulation of T cell proliferation and cytokine production **A.** Flow cytometry plot and graph showing the regulatory effects of CD19^+^CD24^hi^CD38^hi^Bregs on CD3^+^T cells proliferation (NS, no statistical differences). **B.** Graph shows the regulatory effects of Bregs on CD4^+^T cell proliferation (NS, no statistical differences). **C.** Flow cytometry analysis of the inhibitory effects of Bregs on IFN-γ and TNF-α production by CD4^+^T cells. The graphs show the percentage of CD4^+^IFN-γ^+^ and CD4^+^TNF-α^+^among CD4^+^T cells inhibited by the addition of Bregs (NS, no statistical differences, **P < 0.01). **D.** Representative flow cytometry plot showing the Breg regulation of IFN-γ and TNF-α production by CD4^+^T cells in gastric cancer. The graphs show that the percentage of CD4^+^IFN-γ^+^ and CD4^+^TNF-α^+^ of CD4^+^T cells were increased in Bregs-depleted gastric cancer (NS, no statistical differences, *P < 0.05).

To investigate whether increased levels of CD19^+^CD24^hi^CD38^hi^ Breg cells play an immunosuppressive role in gastric cancer, we examined the effect of Bregs on T cell proliferation and inflammatory cytokine production. Sorted CD3^+^T cells and CD4^+^Th cells from HCs stained with carboxyfluorescein succinimidyl ester (CFSE), co-cultured with autologous Bregs (CD24^hi^CD38^hi^) or non-Bregs (CD24^int^CD38^int^), and analyzed via flow cytometry. Neither Bregs nor non-Bregs from HCs suppressed the proliferation of CD3^+^T cells or CD4^+^Th cells (NS, no statistical significance, Figure 4A-4B). To examine the effect of Bregs or non-Bregs on CD4^+^Th cell cytokine expression: sorted CD4^+^Th cells from HCs were co-cultured with autologous sorted Bregs or non-Bregs and intracellular IFN-γ and TNF-α were detected. Bregs suppressed IFN-γ and TNF-α secretion by CD4^+^T cells (*P* < 0.01, Figure 4C), whereas non-Bregs did not significantly inhibit the production of IFN-γ or TNF-α secretion (NS, Figure 4C). Moreover, addition of anti-IL-10 antibody inhibited Bregs from suppressing the secrection of IFN-γ and TNF-α (Figure S5A).

We next explored the regulatory properties of Bregs on CD4^+^Th cells in gastric cancer. Given the limited number of Bregs in GCs, CD24^hi^CD38^hi^Bregs or CD24^int^CD38^int^ non-Bregs were depleted from PBMCs by FACS Aria II. Depletion of Bregs from GCs PBMCs increased the percentage of CD4^+^IFN-γ^+^ and CD4^+^TNF-α^+^ cells among CD4^+^T cells compared to non-depleted PBMCs from the same individuals (*P* < 0.05, Figure 4D). Depletion of non-Bregs did not promote the expression of IFN-γ and TNF-α by CD4^+^T cells (NS, Figure 4D). PBMCs from HCs were also showed an increased percentage of IFN-γ and TNF-α with Breg depletion compared with non-depleted PBMCs (*P* < 0.05, respectively, Figure S5B-C). In summary, CD19^+^CD24^hi^CD38^hi^ Bregs do not suppress T cell proliferation but do inhibit CD4^+^Th cell cytokine production.

### Bregs convert effector T cells into FoxP3-expressing Tregs

**Figure 5:**
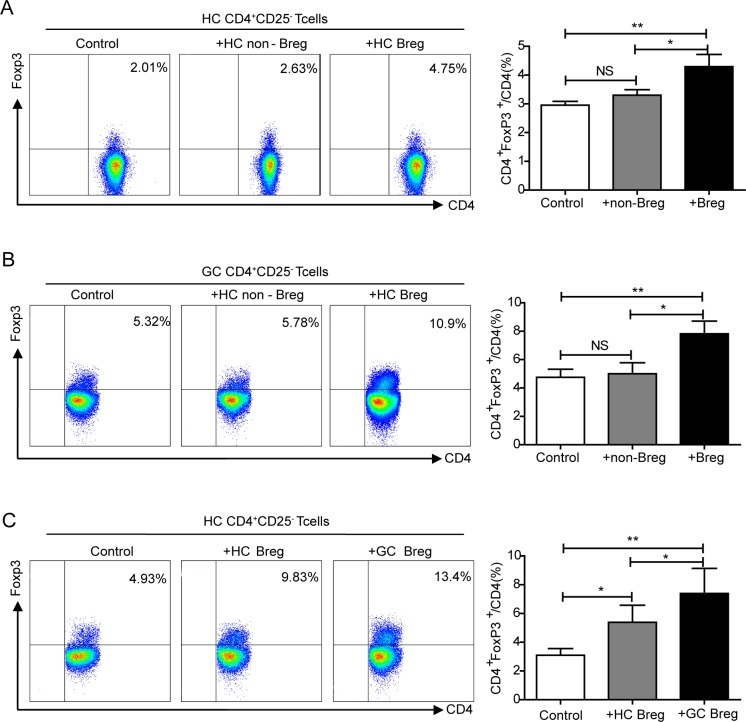
Elucidating the interaction between Bregs and Tregs **A.** Flow cytometry analysis of the interaction between CD19^+^CD24^hi^CD38^hi^Bregs and CD4^+^FoxP3^+^Tregs in HCs. The graph shows that increased FoxP3 expression was detected upon addition of Bregs (NS, no statistical differences, *P < 0.05, **P < 0.01). **B.** Plot and graph illustrating that the percentage of FoxP3 was enhanced by Bregs of GCs (NS, no statistical differences, *P < 0.05, **P < 0.01). **C.** Compared with HCs Bregs, co-culture with Bregs from GCs increased expression of FoxP3 by CD4^+^CD25^−^ effector T cells (*P < 0.05, **P < 0.01).

As already shown, there was a significant positive correlation between Bregs and Tregs in gastric cancer (Figure 3E). We therefore hypothesized that there might be an interaction between CD19^+^CD24^hi^CD38^hi^Bregs and CD4^+^FoxP3^+^Tregs. To assess the effect of Bregs on Tregs, sorted CD4^+^CD25^−^ effector T cells from HCs were cultured with autologous CD24^hi^CD38^hi^Bregs or CD24^int^CD38^int^ non-Bregs for 72 h and FoxP3 labeled CD4^+^T cells were examined via flow cytometry. Compared with controls and non-Bregs, Bregs induced the upregulation of CD4^+^FoxP3^+^Tregs (*P* < 0.01, *P* < 0.05, respectively Figure 5A). We next investigated the regulatory properties of Bregs in gastric cancer. Sorted CD4^+^CD25^−^ effector T cells from GCs were co-cultured with autologous Bregs or non-Bregs. There was a higher increase in FoxP3 expression in CD4^+^CD25^−^T cells co-cultured with Bregs than in those cultured with non-Bregs or controls (*P* < 0.05, *P* < 0.01, respectively Figure 5B). As it was previously reported that Bregs lack suppressive capacity in autoimmune diseases [21, 22], to further determine whether the regulatory properties of Bregs differ between HCs and GCs, sorted CD4^+^CD25^−^ effector T cells from HCs were co-cultured with autologous Bregs or Bregs from GCs. Bregs induced the conversion of CD4^+^CD25^−^ effector T cells to CD4^+^FoxP3^+^Treg cells, and this conversion was strengthened by Bregs from GCs (*P* < 0.05, Figure 5C).

### T cell conversion by CD19^+^CD24^hi^CD38^hi^ Bregs depends upon TGF-β1

**Figure 6:**
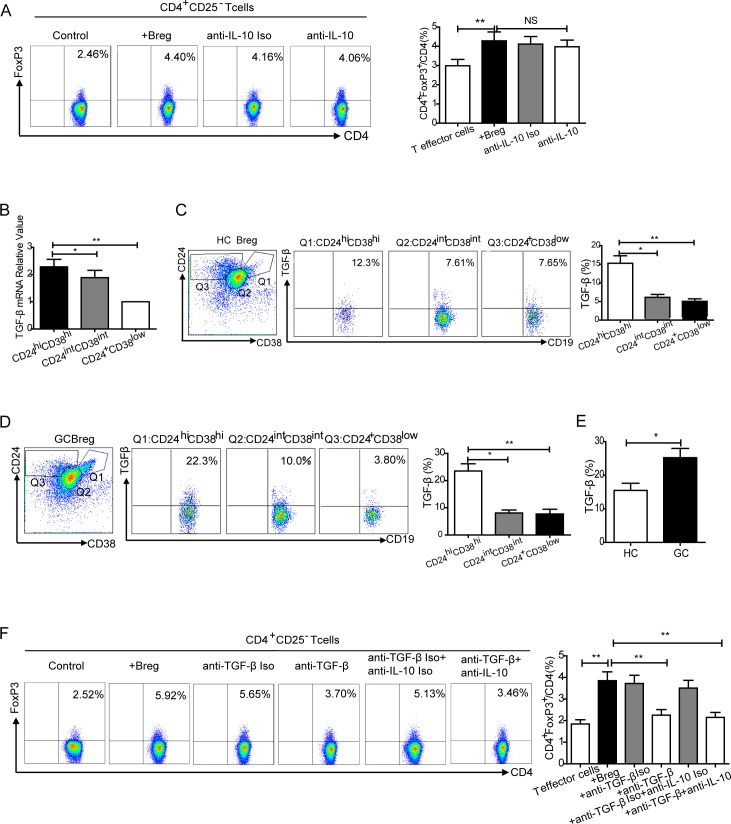
Conversion of effector T cells to Tregs by CD19^+^CD24^hi^CD38^hi^ Bregs **A.** CD4^+^CD25^−^ effector T cells were co-cultured with autologous CD19^+^CD24^hi^CD38^hi^Bregs at 1:1 for 72 h and treated with anti-IL-10 isotype control and neutralizing mAb. There was no obvious inhibition with IL-10 neutralization (NS, no statistical differences, **P < 0.01). **B.** High *TGF-β* mRNA expression was observed in sorted CD19^+^CD24^hi^CD38^hi^Bregs using real-time PCR (*P < 0.05, **P < 0.01). **C.** Representative flow cytometry plot and graph indicating an increased percentage of TGF-β1 in Bregs (CD19^+^CD24^hi^CD38^hi^) compared to two B cell subsets (CD19^+^CD24^int^CD38^int^, CD19^+^CD24^+^CD38^low/−^) from HCs (*P < 0.05, **P < 0.01). **D.** An increased percentage of TGF-β1 was detected in Bregs compared to the other two B cell subsets from GCs (*P < 0.05, **P < 0.01). **E.** Compared with Bregs from HCs, an elevated percentage of TGF-β was detected in Bregs from GCs (*P < 0.05). **F.** Representative flow cytometry plot and graph showing that FoxP3 expression in CD4^+^CD25^−^ effector T cells co-cultured with Bregs was inhibited by TGF-β neutralizing mAb (**P < 0.01).

As shown previously, CD19^+^CD24^hi^CD38^hi^ Bregs produce IL-10. To determine whether IL-10 was involved in the conversion of T cells to Tregs, cells were treated with anti-IL-10 neutralizing monoclonal antibody (mAb) and isotype control. T cell conversion was not effectively inhibited by the neutralization of IL-10 (NS, Figure 6A). As TGF-β was widely believed to be a highly important factor in the development of Tregs, we next quantified *TGF-β* mRNA in sorted CD19^+^CD24^hi^CD38^hi^Bregs, CD19^+^CD24^int^CD38^int^ and CD19^+^CD24^+^CD38^low/−^ B cell subsets from HCs using real-time PCR. Bregs had high *TGF-β* mRNA expression (*P* < 0.05, *P* < 0.01, Figure 6B). Next, the percentage of TGF-β1 positive cells was investigated in Bregs (CD19^+^CD24^hi^CD38^hi^) and two other B cells subsets (CD19^+^CD24^int^CD38^int^, CD19^+^CD24^+^CD38^low/−^) in HCs using flow cytometry. Compared with the other two subsets of B cells, there was an increase in TGF-β1 expression in Bregs (*P* < 0.05, *P* < 0.01, Figure 6C).

We then quantified the percentage of TGF-β1 positive cells in Bregs and the two B cell subsets from GCs. As found in HC cells, there was an increased percentage of TGF-β1 in Bregs from GCs (Figure 6D). Moreover, there was a higher percentage of TGF-β1 positive cells in Bregs from GCs than in HCs (25.2±6.73% *vs*. 15.5±6.78%, *P* < 0.05, Figure 6E). To further determine the role of TGF-β1 during T cell conversion, CD24^hi^CD38^hi^Bregs were co-cultured with CD4^+^CD25^−^ effector T cells and incubated with anti-TGF-β1 neutralizing mAb. T cell conversion was abolished by the addition of TGF-β1 mAb (from 3.85±1.08% to 2.25±0.68%, *P* < 0.01, Figure 6F). These results suggest T cell conversion to CD4^+^FoxP3^+^Tregs by Bregs is dependent on TGF-β1.

## DISCUSSION

Bregs play a key role in inflammation, autoimmune diseases, and other inflammatory disease [9, 16, 18, 20-23, 27-34]. However, little is known about the potential roles of Bregs in the development of human tumors. We are the first to investigate the characterization of Bregs in human gastric cancer and reveal their possible roles and mechanisms of action. In this study, we determined that increased CD19^+^CD24^hi^CD38^hi^ Bregs in gastric cancer possess a robust immune regulatory capacity to produce IL-10 and TGF-β1. Increased CD19^+^CD24^hi^CD38^hi^Bregs positively correlate with levels of CD4^+^FoxP3^+^Tregs in gastric cancer. Moreover, Bregs play a significant immunosuppressive role in gastric cancer, not only by inhibiting CD4^+^Th cell cytokine production, but also by converting CD4^+^CD25^−^ effector T cells to CD4^+^FoxP3^+^Tregs in a TGF-β1- dependent way. Our results provide important support for an essential role of information for Bregs in human gastric cancer.

As shown in previous studies, there is no consensus regarding the most appropriate combination of Breg-linked markers, and therefore different research teams have used diverse surface and intracellular markers of regulatory B cells [13-21, 24, 25, 37]. Approximately 21 biomarkers in mouse and human Breg cells [12] and some other markers in murine tumor models [42] have been reviewed. However, Breg cell function and cell sorting analysis depend on the type and number of markers used; therefore, the most appropriate markers for Bregs in human gastric cancer had to be confirmed first. Although there are still no specific markers or unique nuclear transcription factors for Bregs, emerging evidence has suggested that IL-10 is the defining trait of Bregs [9, 41]. Our results demonstrate that CD19^+^CD24^hi^CD38^hi^ B cell subsets possess a robust immune regulatory capacity to produce IL-10 in gastric cancer, which is consistent with a previous report in SLE and includes most CD5^+^CD1d^hi^ B cells [21]. Our results also support the immunoregulatory phenotypes of Bregs in both mice models and humans reported previously. Our phenotypic research on Bregs in gastric cancer may provide important data for defining Bregs and provide a reference for use in research on human tumors.

In our study, we detected significantly increased CD19^+^CD24^hi^CD38^hi^ Bregs in gastric cancer patients. The enhanced percentage of Bregs may invole in effector immune responses and could have a significant impact on the development of gastric cancer. As we all know, CD4^+^Th cells are essential to the immune response of anti-tumor and Th1 cells [43, 44], a predominant Th cell subtype characterized by IFN-γ and TNF-α secretion. We showed that there is no noticeable inhibition of CD3^+^T cells or CD4^+^Th cells proliferation by Bregs, which is consistent with a previous report in mice [45] and MS patients [28]. Although Bregs did not suppress the expansion of T cells *in vitro*, their roles in cytokine production cannot be ruled out. We observed that Bregs from gastric cancer do inhibit the production of IFN-γ and TNF-α. However, inconsistent with our present results, it has been reported that Bregs failed to suppress CD4^+^T cell cytokine production in SLE [21]. Perhaps some functions of Bregs are impaired in autoimmune diseases.

As our previous studies reported an enhanced frequency of Tregs in gastric cancer [5-7] and Tregs hamper anti-tumor immune responses effectively [46-49], we wondered whether there was a correlation between Bregs and Tregs. As expected, we found a positive correlation between CD24^hi^CD38^hi^Bregs and CD4^+^FoxP3^+^Tregs in gastric cancer. We also showed that, in addition to IL-10, CD24^hi^CD38^hi^Bregs produce TGF-β1. Furthermore, CD24^hi^CD38^hi^Bregs convert CD4^+^CD25^−^ effector T cells into CD4^+^FoxP3^+^ Tregs, which are dependent on TGF-β1 in gastric cancer. Our results are in contrast with another study in RA, which showed that Bregs could not promote Treg conversion in RA patients [22]. This functional discrepancy of Breg cells may be attributed to the different diseases states. In addition, due to the lack of consistent markers for Bregs, the cells identified as Bregs in other studies, both *in vitro* and in mouse model systems, may not represent the same cell types, which could also explain the discrepancies.

Collectively, our results suggest that Breg cells might be involved in gastric cancer immune escape, promoting cancer progression. The enhanced suppressive capacity of Bregs in gastric cancer disturbs the balance of the tumor-protective and tumor-promoting functions. This acceleration of tumor progression accelerating function was also recently reported in Bregs from hepatocellular carcinoma [50]. Importantly, all of our functional studies were conducted with Bregs in peripheral blood from healthy controls or gastric cancer patients. The function of Bregs from tumor tissues was not examined because the MFI of CD24 was decreased in IL-10^+^ B cells, less than in IL-10^−^ B cells from tumor (*P* < 0.05, Figure S2B) and few CD24^hi^CD38^hi^Bregs were detected in tumor tissues (Figure S6), as confirmed by newly published research [51].

**Figure 7:**
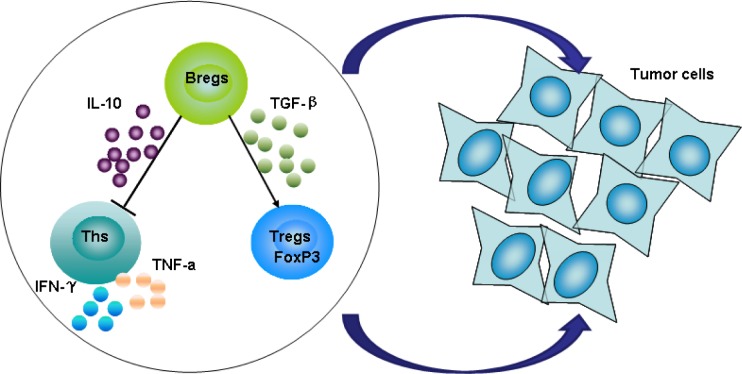
Hypothetical model for the function of CD19^+^CD24^hi^CD38^hi^ Bregs in the development of gastric cancer Regulatory B cells secrete the inhibitory cytokine IL-10, suppressing the generation of IFN-γ and TNF-α by CD4^+^Th cells, while also producing TGF-β to promote regulatory T cell conversion. These factors may impair the anti-tumor response, promoting immune escape in the tumor microenvironment in gastric cancer.

Collectively, as summarized in Figure 7, our findings demonstrate that regulatory B cells are important for gastric cancer immune escape by suppressing the generation of IFN-γ and TNF-α from CD4^+^Th cells and producing TGF-β1 to promote regulatory T cell conversion. These actions of Bregs, all of which impair the anti-tumor response, promote immune escape in the tumor microenvironment. Of course, other possible role of Bregs in gastric cancer development and underlying mechanisms should be explored further. Considering all the data presented here, CD19^+^CD24^hi^CD38^hi^ regulatory B cells may play an important role in the development of gastric cancer. This research may provide new insights into the biology of Breg functions as well as new clues to novel treatment for gastric cancer and, possibly, other human cancers.

## MATERIALS AND METHODS

### Patients and controls

Patients with gastric cancer (*N* = 107, 25 females and 82 males, mean age = 65.6 years, age range from 33 to 84 years) without previous treatments were enrolled in the study upon giving informed consent. According to the TNM classification for gastric cancer (International Union Against Cancer, UICC) (2010, 7^th^edition), the patients were divided into four stages (Supplementary Table 1). Closely age-matched, healthy subjects (*N* = 45, 16 male and 29 females, mean age = 63.6 years, age range from 35 to 80 years) were recruited as healthy controls. Tumor tissues, peritumoral tissues (at least 2 cm distant from the tumor site) and normal tissues (at least 4 cm distant from the tumor site) were obtained intraoperatively from 45 patients. This study was approved by the ethical committee of Xinhua Hospital affiliated to Shanghai Jiao Tong University School of Medicine.

### Cell preparation

Cell preparation was performed as described in the Supplementary Information.

### Multicolor flow cytometry analysis

The following antibodies were used: anti-human CD19, CD21, CD38, TBNK 6-Color Kit and isotype controls (BD Biosciences), CD24, CD1d, IL-10, FoxP3 (eBiosciences), CD3, CD4, CD5, CD10, CD22, CD25, CD45, IFN-γ, TNF-α, IL-4 and isotype controls (all from Beckman Coulter), IgM (DAKO), TGF-β1 and isotype controls (Biolegend). Cell staining and flow cytometry analysis were performed according to routine laboratory methods, as described in the Supplementary Information. Stained cells were analyzed via flow cytometry analysis using a FACS Canto II (BD Biosciences). Data were analyzed using FlowJo software (Tree Star, Ashland, OR).

### Cells sorting

After staining with surface markers, CD19^+^B cells, CD3^+^T cells, CD4^+^T cells, CD4^+^CD25^−^T effector cells, CD19^+^CD24^hi^CD38^hi^, CD19^+^CD24^int^CD38^int^, CD19^+^CD24^+^CD38^low/−^B subset cells were sorted from healthy controls and gastric cancer patients using FASC Aria II (BD Biosciences) according to the instructions.

### Co-culture and neutralization assay of Bregs

Cell co-culture and flow cytometry analysis were performed as described in the Supplementary Information. The neutralization of anti-IL-10 antibody (R&D, final concentration of 5 μg/ml) and isotype control antibody (R&D, mouse IgG2B) or anti-TGF-β1 neutralizing antibody (R&D, final concentration of 4 μg/ml) and isotype control antibody (R&D, mouse IgG1) was performed during co-culture experiment.

### Immunohistochemistry and confocal microscopy analysis

Tumor tissues and peritumoral tissues were obtained intraoperatively from 45 patients. Immunohistochemical staining and immunofluorescence were performed as previously described [35]. Details are described in the Supplementary Information.

### RNA extraction and real-time PCR

CD19^+^CD24^hi^CD38^hi^, CD19^+^CD24^int^CD38^int^ and CD19^+^CD24^+^CD38^low/−^ B subset cells were sorted, and total RNA was extracted from the sorting B cell subsets using RNA Extracting mini Kits (Qiagen). After RT-PCR (Takara), real-Time PCR (Takara) was performed according to kit instructions. Relative expression was determined using the 2^−ΔΔCt^ method with averaged relative levels of GAPDH used for normalization. The sequences of primers are listed in Supplementary Table 2.

### Cytokine detection via ELISA assay

Serum from healthy controls and gastric cancer patients was stored at −80°C. Serum IL-10 levels in healthy control and gastric cancer patients were measured using a commercially available ELISA system (R&D) following the instructions of the manufactures. The reactions were quantified via spectrophotometry using a microplate reader (SYNERGY2, BioTek, USA).

### Statistical analysis

All data are shown as mean ± SD. Statistical analyses were performed with GraphPad Prism 5.0 using linear regression, t test (Paired t test or Mann-Whitney Test, two groups) or two-way ANOVA for all comparisons described in the study, and differences were considered significant at *P* < 0.05 (*P < 0.05, **P < 0.01 and ***P < 0.001).

## SUPPLEMENTARY MATERIAL FIGURES AND TABLES


