# Supplementary material for: CD19+CD24hiCD38hiBregs involved in downregulate helper T cells and upregulate regulatory T cells in gastric cancer
Source: Oncotarget. 2015 Sep 10;6(32):33486–99. doi: 10.18632/oncotarget.5588 (PMC4741780; doi:10.18632/oncotarget.5588)
Supplement: Supplementary file 1 [file oncotarget-06-33486-s001.pdf]

CD19^{+}CD24^{hi}CD38^{hi}Bregs involved in downregulate helper T cells and upregulate regulatory T cells in gastric cancer

Supplementary Material

Cell Preparation

Peripheral blood samples were collected from gastric cancer patients prior to surgery and from age-matched healthy controls. Peripheral blood mononuclear cells (PBMCs) were isolated by ficoll density gradient centrifugation (Norway). Fresh tumor tissues, autologous peritumoral tissues, and normal tissues were washed with RMPI-1640 (Hyclone), cutted into small pieces and digested with 0.1% Collagenase Ⅳ (Gibco), 0.002% DNAse Ⅰ (Sigma), and 0.01% Hyaluronidase (Sigma) at 37°C and 80 rpm for 40 min. Dissociated cells were filtered through a 120 μm mesh. Tumor infiltrating lymphocytes (TIL) and non-tumor infiltrating lymphocytes (NIL) were isolated after ficoll density gradient centrifugation.

Cells staining and flow cytometry analysis

B cell phenotypic analysis was performed with PBMCs (1*10^6 cells/ml) and fluorescently conjugated monoclonal antibodies. For intracellular IL-10 expression studies, PBMCs or TIL and NIL were resuspended (1*10^6cells/ml) in DMEM media (Gibico) containing 10% Fetal Bovine Serum (Gibico) and 50 U/ml antibiotics (Gibico), followed by stimulation with 100 nM CpG ODN2006 (InVivogen) for 72 h; PMA (50 ng/ml), ionomycin (500 ng/ml) (all from Sigma) and GolgiStop (Monensin, BD Bioscience) were added 5 h before the end of the culture. Following B cell surface staining, cytoplasmic IL-10 expression was analyzed using a Cytofix/Cytoperm kit (BD Biosciences) according to the manufacturer’s instructions, staining with isotype control or anti-human IL-10. For intracellular TGF-β expression studies, following B cell surface staining, cytoplasmic TGF-β expression was analyzed using a Cytofix/Cytoperm kit according to kit instructions and staining with isotype control or anti-human TGF-β.

Analyses of T cells were performed with PBMCs (1*10^6cells/ml) using fluorescently conjugated antibodies CD3, CD4, CD25, and CD45. For intracellular staining, cells were

resuspended ($1*10^6$ cells/ml) in 1640 media containing 10% Fetal Bovine Serum and antibiotics before stimulation with anti-human CD3 (10 μg/ml), anti-human CD28 (10 μg/ml) (all from eBiosciences) and IL-2 (100 U/ml, Peprotech) for 72 h. PMA, ionomycin and GolgiStop were added 5 h before the end of the cultures. Following T cell surface staining, intracellular IFN-γ, IL-4, FoxP3 expression was analyzed using a Cytofix/Cytoperm kit or FoxP3 FIX/PERM kit (eBiosciences) according to the manufacturer's instructions and staining with isotype control or intracellular antibodies.

Cells co-culture

For T cell proliferation, purified (>95%, $2*10^5$ cells/96 U well) $CD3^+$T cells and $CD4^+$Th cells were first stained with carboxyfluorescein succinimidyl ester (CFSE, Invitrogen) and then co-cultured with or without autologous $CD19^+CD24^{hi}CD38^{hi}$ Bregs or $CD19^+CD24^{int}CD38^{int}$ non-Bregs at 1:1 in the presence of anti-CD3/CD28 (10 μg/ml) and IL-2 (100 U/mL) for 7 d.

For T cell intracellular staining, purified (>95%, $2*10^5$ cells/ 96 U well) $CD3^+$T cells and $CD4^+$Th cells were co-cultured with Bregs or non-Bregs at 1:1 in the presence of anti-CD3/CD28 and IL-2 for 72 h. PMA and ionomycin and GolgiStop were added 5 h before the end of the cultures.

For Tregs conversion, purified (>95%, $2*10^5$ cells/96 U well) $CD4^+CD25^-$T effector cells were co-cultured with Bregs or non-Bregs at 1:1 in the presence of anti-CD3/CD28 and IL-2 for 72 h; PMA, ionomycin and GolgiStop were added 5 h before the end of the cultures.

Immunohistochemistry and confocal microscopy analysis

For immunohistochemical staining, sections were stained with mouse anti-human CD19 (at a 1:100 dilution, DAKO) and horseradish peroxidase (HRP)-labeled anti-mouse secondary antibody (Invitrogen), according to kit instructions. Images were viewed and assessed via scanning microscopy (DM2000, Leica) and analyzed using LAS AF3.8 software.

For immunofluorescence, sections were stained overnight at 4°C with mouse anti-human CD19 (at a 1:100 dilution, Abcam) and rabbit anti-human IL-10 (at a 1:100 dilution, Abcam). After washing in PBS, the samples were incubated with secondary antibodies: AF488-conjugated goat anti-mouse IgG at a 1:200 dilution and AF594-conjugated goat anti-rabbit IgG at a 1:200 dilution (all from Invitrogen) for 30 minutes at room temperature before washing with PBS. Images were viewed and assessed using a scanning confocal microscopy (Leica TCS SP5) and analyzed by Leica LAS AF software.

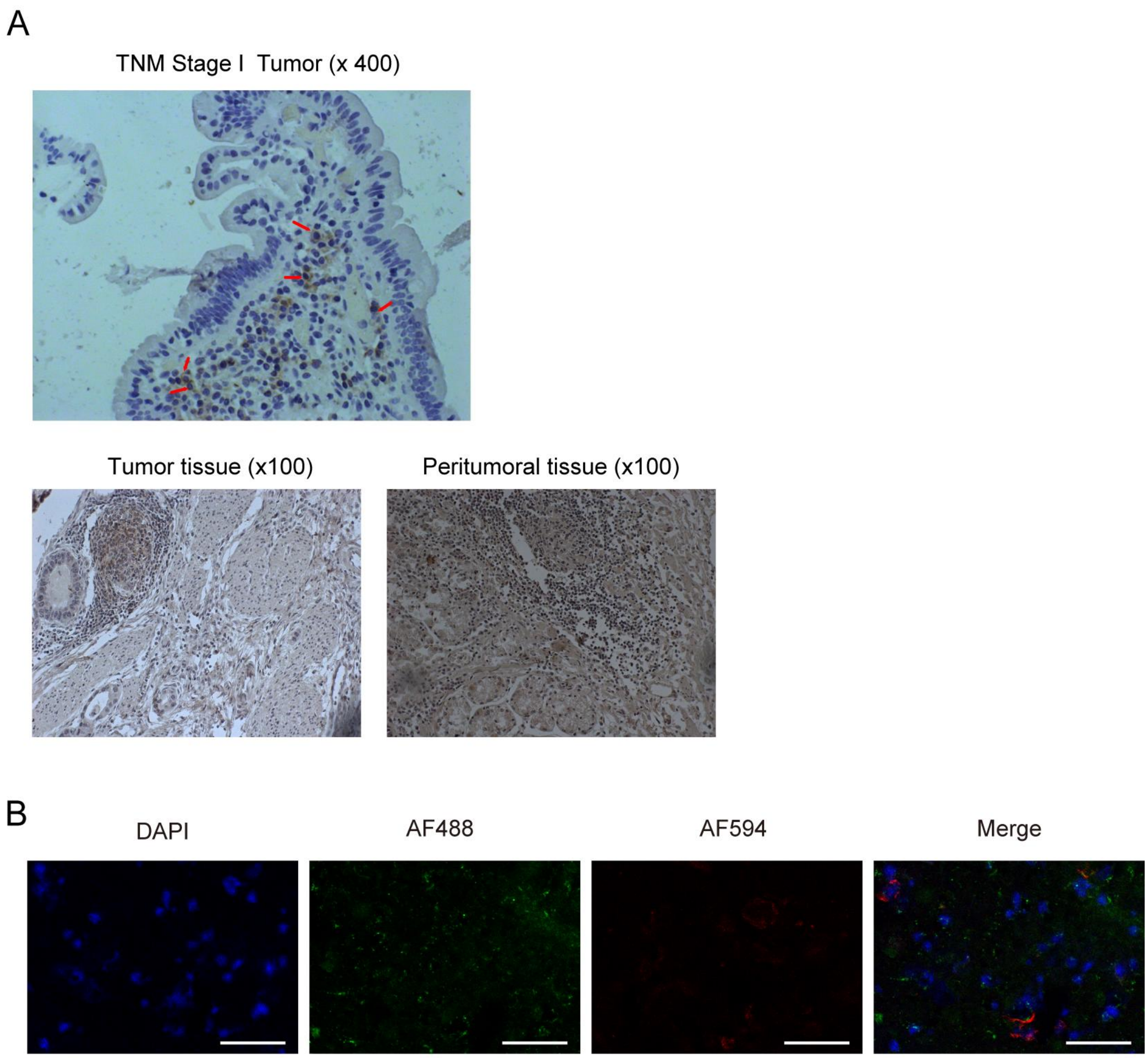

Figure S1. Immunohistochemical staining and confocal microscopy were performed in tumor tissues from gastric cancer. A. Immunohistochemical photos showing the distribution of $CD19^{+}$B cells in paraffin-embedded tumor tissues and peritumoral tissues. B. Split image displaying labelling of DAPI (blue) and CD19 (green) and IL-10 (red) and merged figure in tumor tissues from gastric cancer. Scale bar is 25 μm.

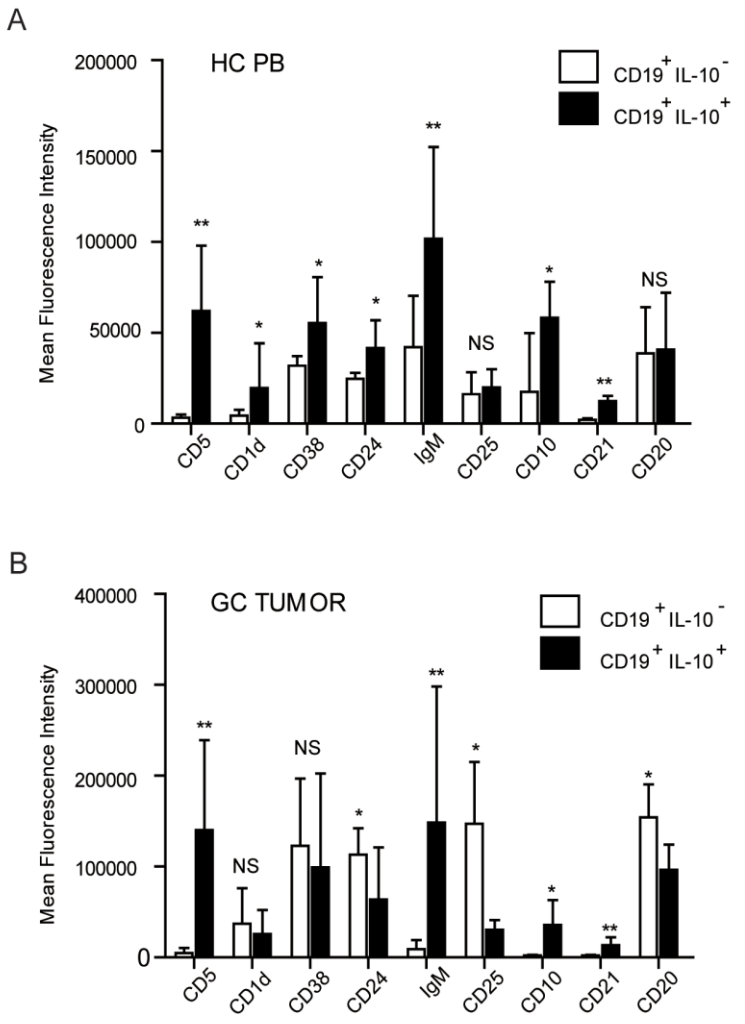

Figure S2. Flow cytometry analysis of IL-10-producing Bregs both in PBMC from healthy controls and tumor tissues from gastric cancer. A. The phenotype of IL-10-producing Bregs from healthy control was analyzed using flow cytometry. The histogram depicts mean fluorescence intensity (MFI) of different cell surface makers expressed in IL-10^{+} (black) and IL-10^{-} (blank) CD19^{+} B cells. Increased MFI was detected on IL-10^{+} B cells compared to IL-10^{-} B cells (*P<0.05, **P<0.01, NS, no statistical differences). B. The phenotype of IL-10-producing Bregs from tumor tissues as analyzed using flow cytometry. The histogram depicts MFI of different cell surface makers expressed in IL-10^{+} (black) and IL-10^{-} (blank) CD19^{+} B cells (*P<0.05, **P<0.01, NS, no statistical differences). Decreased MFI of CD24 was detected on IL-10^{+} B cells compared to IL-10^{-} B cells (*P<0.05).

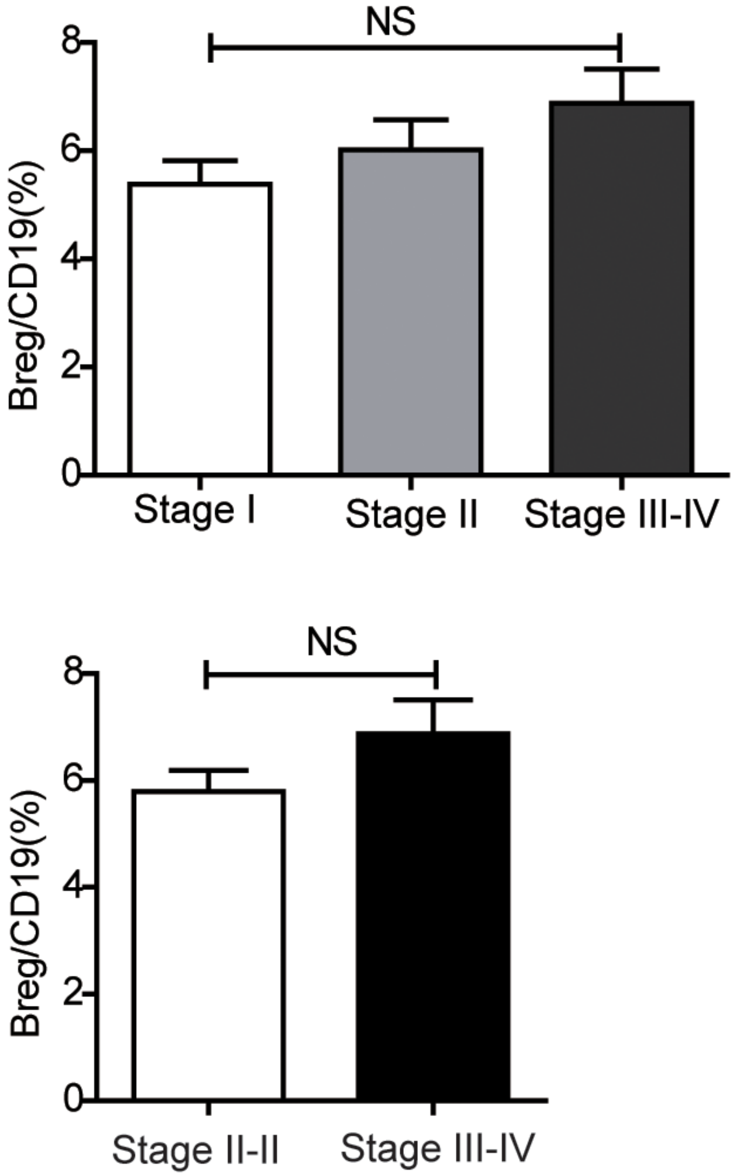

Figure S3. Flow cytometry analysis of the percentage of CD19^{+}CD24^{hi}CD38^{hi} Bregs gated in CD19^{+} B lymphocytes in PBMCs from gastric cancer. There were no significant differences among the four TNM stages (NS, no statistical differences), where Bregs in patients with advanced stages of gastric cancer (Ⅲ–Ⅳ) were higher than in patients with early stages (Ⅰ-Ⅱ), although there were no significant differences (NS, no statistical differences).

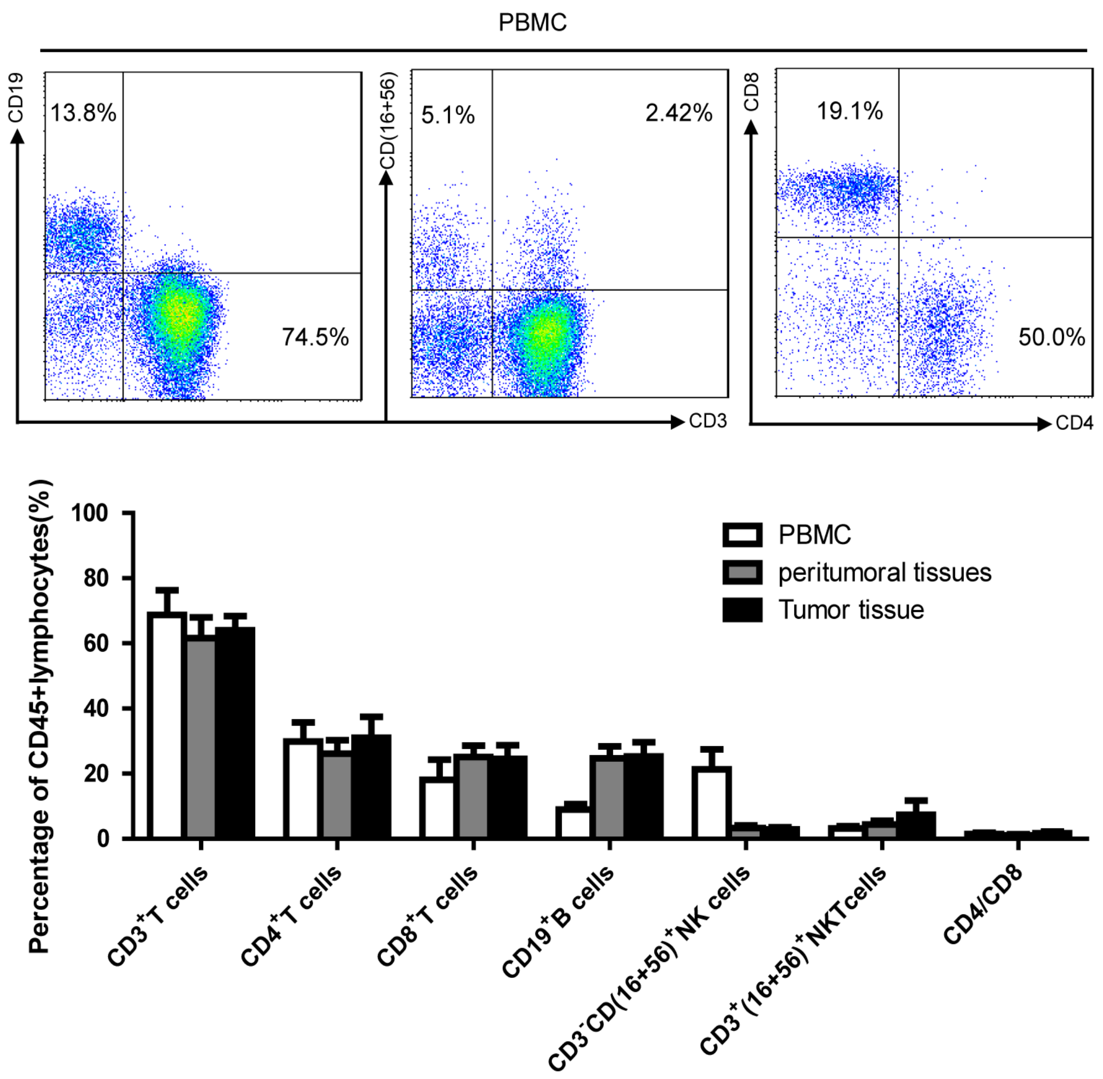

Figure S4. Flow cytometry plot and graph showing the percentage of different cell subsets gated in $CD45^+$ lymphocytes in PBMC, peritumoral tissues and tumor tissues from gastric cancer, including $CD3^+$T cells, $CD3^+CD4^+$Th cells, $CD3^+CD8^+$Tc cells, $CD19^+$B cells, $CD3^-(CD16+56)^+$NK cells, $CD3^+(CD16+56)^+$NKT cells and the ration of CD4/CD8.

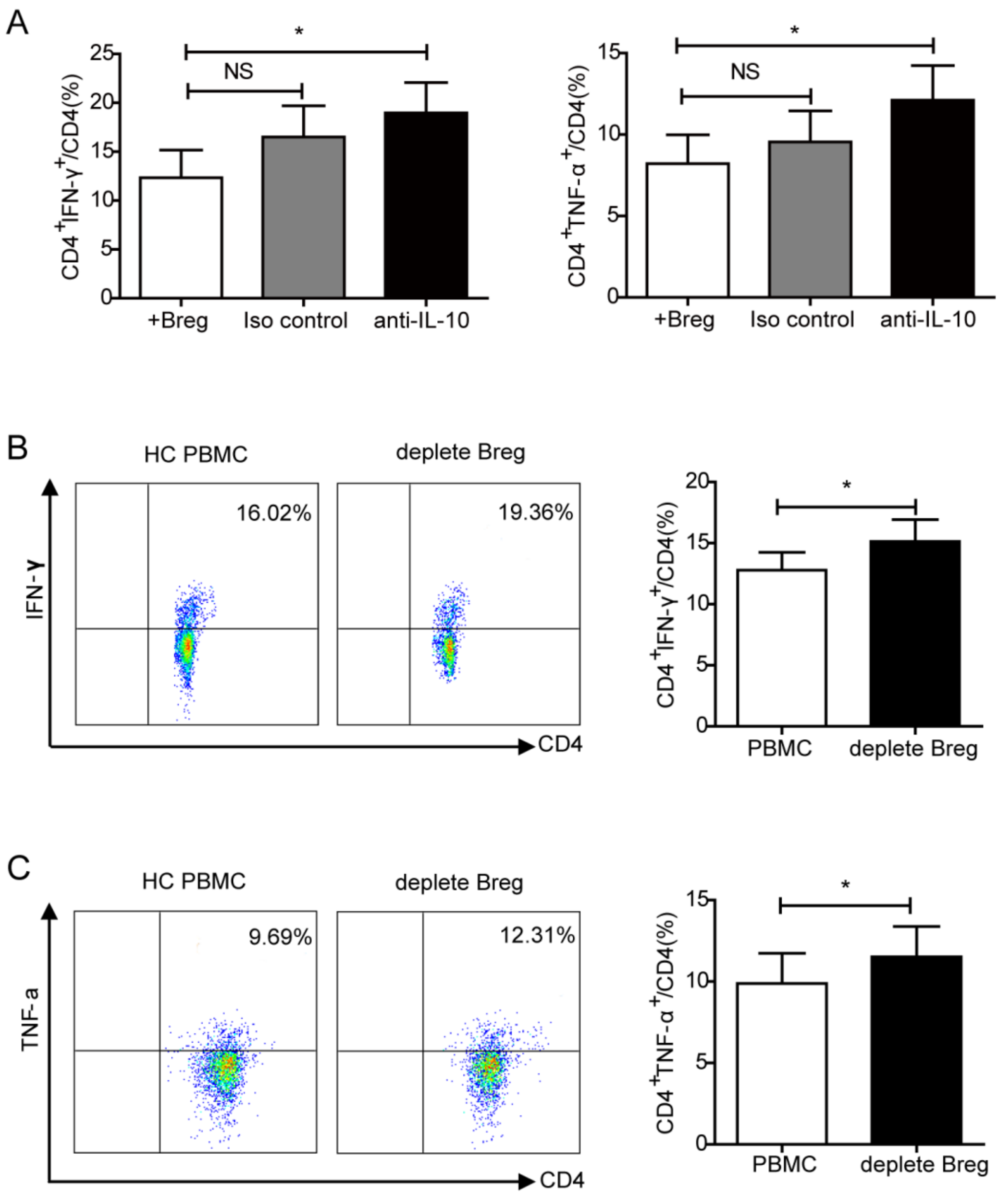

Figure S5. Healthy controls CD19$^+$CD24^{hi}CD38^{hi} Bregs suppress IFN-γ and TNF-α production acting through IL-10. A. The addition of anti-IL-10 neutralization antibody inhibited Bregs from suppressing the percentage of IFN-γ and TNF-α production by CD4$^+$T cells (*P<0.05). B. Depletion of CD19$^+$CD24^{hi}CD38^{hi} Bregs from healthy controls increased the percentage of IFN-γ produced by CD4$^+$T cells compared to controls (*P<0.05). C. Depletion of CD19$^+$CD24^{hi}CD38^{hi} Bregs from healthy controls increased the percentage of CD4$^+$TNF-α$^+$ T cells compared to controls (*P<0.05).

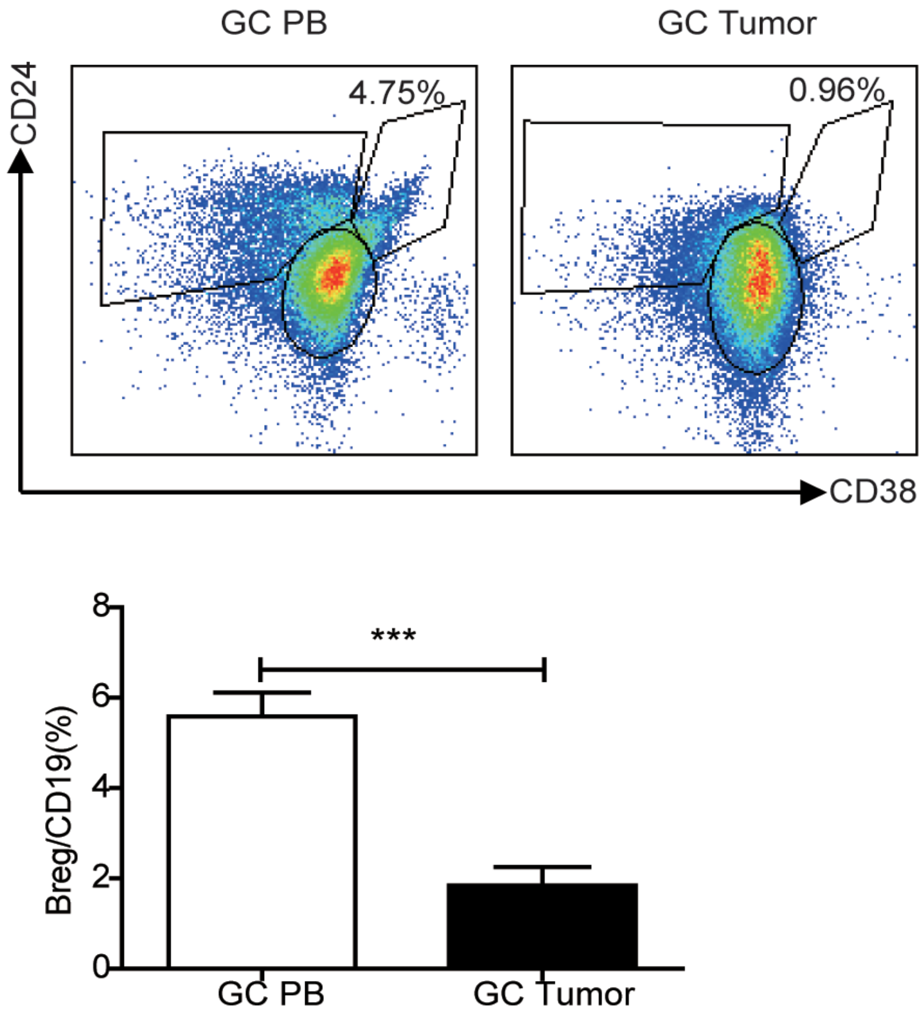

Figure S6. Flow cytometry analysis of the percentage of $CD19^{+}CD24^{hi}CD38^{hi}$ Bregs gated in $CD19^{+}$ B cells both in PBMCs and tumor tissues from gastric cancer. Few $CD19^{+}CD24^{hi}CD38^{hi}$ Bregs were detected in tumor tissues in gastric cancer. Plot and graph showing the percentage of $CD19^{+}CD24^{hi}CD38^{hi}$ Bregs in tumor tissues was far less than that in PBMCs (****P*<0.001).

Supplementary Table 1. Characteristics of the healthy controls and gastric cancer patients

	Healthy control	Gastric cancer
number	40	107
Age(year)(mean)	35-80(63.6)	33-84(65.6)
>=60	22	80
<60	18	27
Gender(F/M)	12/28	25/82
TNM stage		
I	0	18
II	0	40
III	0	47
IV	0	2

Supplementary Table 2. Nuclear sequences of primers used in real-time PCR

Gene	Sequence
GAPDH	5’-ATTCCACCCATGGCAAATTC-3’
	5’- GCATCGCCCCACTTGATT -3´
TGF-β1	5’-AGCGACTCGCCAGAGTGGTTA-3’
	5’-GCAGTGTGTTATCCCTGCTGTCA - 3’
